# Activated dendritic cells modulate proliferation and differentiation of human myoblasts

**DOI:** 10.1038/s41419-018-0426-z

**Published:** 2018-05-10

**Authors:** Leandro Ladislau, Débora M. Portilho, Tristan Courau, Alhondra Solares-Pérez, Elisa Negroni, Jeanne Lainé, David Klatzmann, Adriana Bonomo, Yves Allenbach, Olivier Benveniste, Ingo Riederer, Wilson Savino, Vincent Mouly, Gillian Butler-Browne, Claudia F. Benjamim

**Affiliations:** 1 Institute of Biophysics Carlos Chagas Filho, Federal University of Rio de Janeiro, Rio de Janeiro, Brazil; 2 Institut de Myologie, INSERM U974, Sorbonne Université, Paris, France; 3 Immunology-Immunopathology-Immunotherapy, INSERM U959, Sorbonne Université, Paris, France; 4 Laboratory on Thymus Research, Oswaldo Cruz Institute, Fiocruz, Rio de Janeiro, Brazil; 5 National Institute of Science and Technology on Neuroimmunomodulation, Rio de Janeiro, Brazil

## Abstract

Idiopathic Inflammatory Myopathies (IIMs) are a heterogeneous group of autoimmune diseases affecting skeletal muscle tissue homeostasis. They are characterized by muscle weakness and inflammatory infiltration with tissue damage. Amongst the cells in the muscle inflammatory infiltration, dendritic cells (DCs) are potent antigen-presenting and key components in autoimmunity exhibiting an increased activation in inflamed tissues. Since, the IIMs are characterized by the focal necrosis/regeneration and muscle atrophy, we hypothesized that DCs may play a role in these processes. Due to the absence of a reliable in vivo model for IIMs, we first performed co-culture experiments with immature DCs (iDC) or LPS-activated DCs (actDC) and proliferating myoblasts or differentiating myotubes. We demonstrated that both iDC or actDCs tightly interact with myoblasts and myotubes, increased myoblast proliferation and migration, but inhibited myotube differentiation. We also observed that actDCs increased HLA-ABC, HLA-DR, VLA-5, and VLA-6 expression and induced cytokine secretion on myoblasts. In an in vivo regeneration model, the co-injection of human myoblasts and DCs enhanced human myoblast migration, whereas the absolute number of human myofibres was unchanged. In conclusion, we suggest that in the early stages of myositis, DCs may play a crucial role in inducing muscle-damage through cell–cell contact and inflammatory cytokine secretion, leading to muscle regeneration impairment.

## Introduction

The immune system has evolved allowing complex organisms to be protected against pathogens while maintaining health, including avoidance of harmful self-recognition. However, during autoimmune diseases, the balance between these major biological functions is modified and inflammation together with immune activation persist in the absence of an identified infection or challenge^1^. Idiopathic Inflammatory Myopathies (IIMs) are a group of heterogeneous autoimmune diseases characterized by muscle weakness and inflammatory infiltration in skeletal muscle with limited therapy^2^. They include polymyositis (PM), dermatomyositis (DM), necrotizing autoimmune myopathy (IMNM), and sporadic inclusion body myositis (sIBM)^3^. Steroids and immunosuppressants are effective for PM and DM, but not for IBM; however, these drugs just delay the disease evolution and also present severe side effects. The inflammatory infiltrates are mainly composed of mononuclear cells that include T cells, macrophages and dendritic cells (DCs)^4–6^. The exact mechanisms that trigger and maintain IIMs are poorly understood, but it is known that they exhibit different physiopathology: in IBM CD8^+^ T cell mediate cytotoxicity; in DM high levels of type I interferon correlated with severity;^7^ and in IMNM the muscle lesion is due to the presence of auto-antibodies^8^. Auto-antibodies have been reported in several IIMs suggesting an important implication^9^.

The presence of DCs in the perimysium and perivascular areas in skeletal muscle have been demonstrated in several myopathies including DM, PM, and IBM^4,6,10–17^. The DCs described in skeletal muscle are mainly the plasmacytoid dendritic cells (pDCs), which can present tolerogenic or anti-inflammatory response, although myeloid DCs can also be found in the muscles of IIMs patients^6,7,11^, which are defined as classic DCs. However, the DCs characterizations are controversial due to the small number of samples and limited detection techniques^18–23^. In general, more activated DCs (actDCs) rather than immature DCs (iDC) have been observed in DM and PM muscle biopsies^11,24^. In addition, the detection of CCR7^+^ DCs within muscle tissue raised the hypothesis of an in situ activation of these cells^4^, although this remains to be fully demonstrated. The chemokine receptor CCR7 is the master mediator for DC and T cell migration into lymphoid organs, allowing the contact of these CCR7^+^ cells and the triggering of the immune response.

The presence of inflammatory cells and cytokine release in the skeletal muscle tissue in IIMs^25,26^ have been suggested to be involved both in the muscle atrophy and necrosis/regeneration^27^ observed in these myopathies. Following muscle necrosis, muscle-derived progenitor cells^28^, called satellite cells, become activated, proliferate, migrate, and differentiate into myotubes^29,30^ repairing the damaged muscle fibers. The machinery necessary for myoblast proliferation and differentiation into new muscle fibers is finely regulated^31–33^. In particular, myogenic regulatory factors (MRF), such as myoD and myogenin control the commitment and differentiation of myoblasts after tissue damage^34–36^. This step is critical for the new fiber formation and muscle repair.

Although DCs may be involved in IIMs, their exact role in the pathophysiology of this disease remains unknown. We hypothesized that during early events in myositis, DCs in the muscle tissue may trigger and feed the inflammatory response, consequently leading to muscle atrophy and necrosis, thus impairing muscle regeneration. Due to the absence of a good experimental model for myositis, we developed a co-culture protocol with human myoblasts/myotubes and DCs to investigate cell–cell interactions, cytokine release and myoblast proliferation and differentiation. In addition, using a xenotransplantation model with immunodeficient mice, we evaluated the effect of human DCs upon human myoblast regeneration.

## Results

### Tight interactions between myoblasts or myotubes with actDCs

According to our hypothesis that DCs modulate myoblast function in IIMs, we performed in vitro experiments to investigate specific cell–cell interactions. Monocytes from peripheral blood (healthy donors) were differentiated into immature and steady state DCs (iDC). After 7 days of differentiation into DC’s, the iDC lose their adherent skills and become round, floating, and refringent cells^37^. Once activated, the actDCs take on a more spread and stellate shape and express co-stimulatory molecules^38^.

**Fig. 1:**
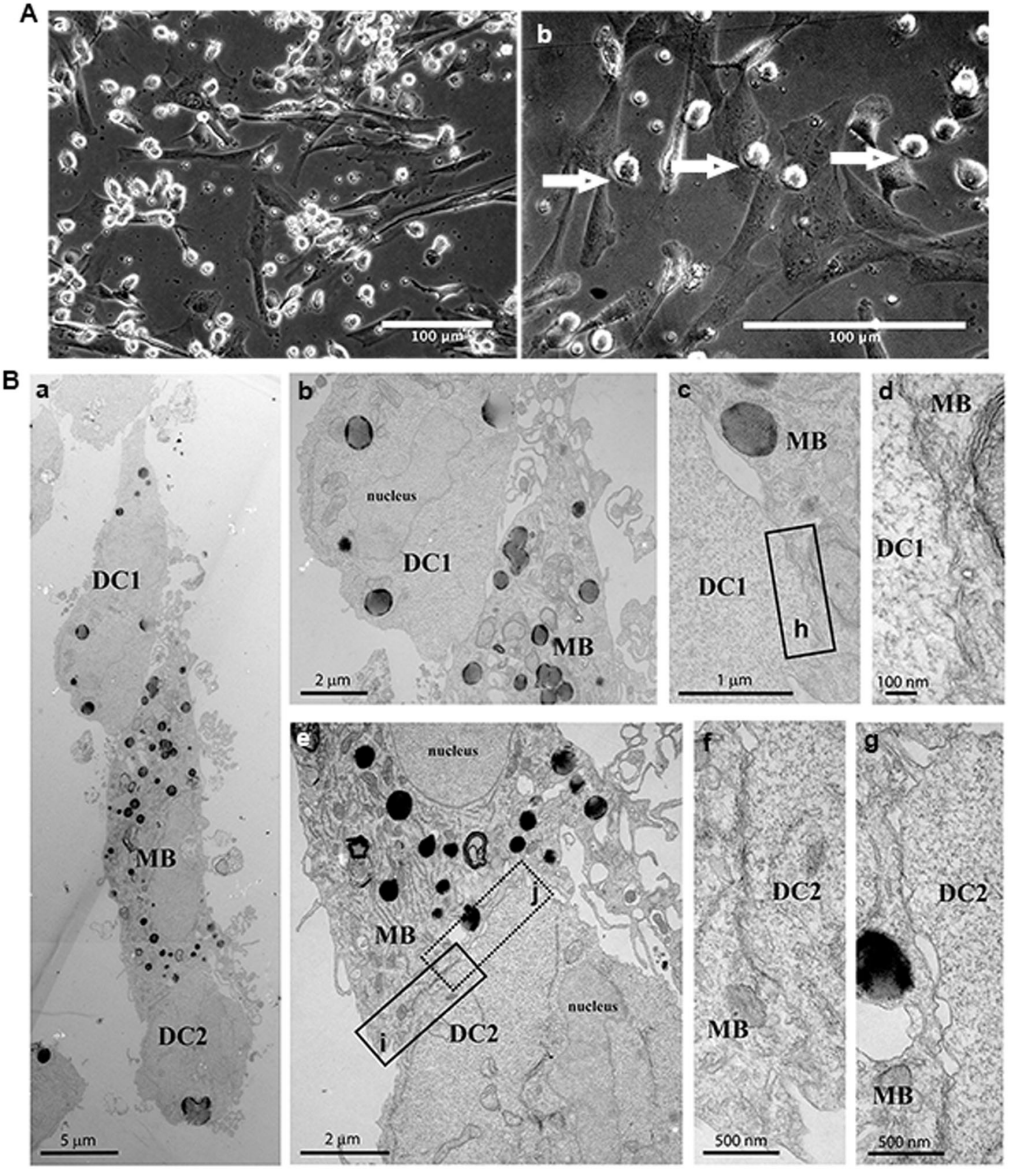
Close contact during co-culture between DCs and myoblasts or myotubes. **A** Phase contrast microscopy of myoblasts co-cultured with actDCs (LPS 100 ng/mL) (**a** and **b**) for 48 h. **a** represents a low magnification and **b** a higher magnification. Bars represent 100 μm. **B** Transmission electron microscopy of myoblasts and actDCs (LPS 100 ng/mL) co-cultured in proliferation medium for 48 h (**a**). Different magnifications of the close contact area of myoblast (MB) and DC1 (**b**, **c**, **d**), while (**d**) is the high magnification of the rectangle (**h**) in (**c**). Different magnifications of the close contact area of myoblast (MB) and DC2 (**e**, **f**, **g**), while (**f**, **g**) are high magnifications of the rectangles (**i**, **j**) seen in (**e**), respectively. Bars represent 5, 2, 1, 100, and 500 nm. Data are representative of three independent experiments

**Fig. 2:**
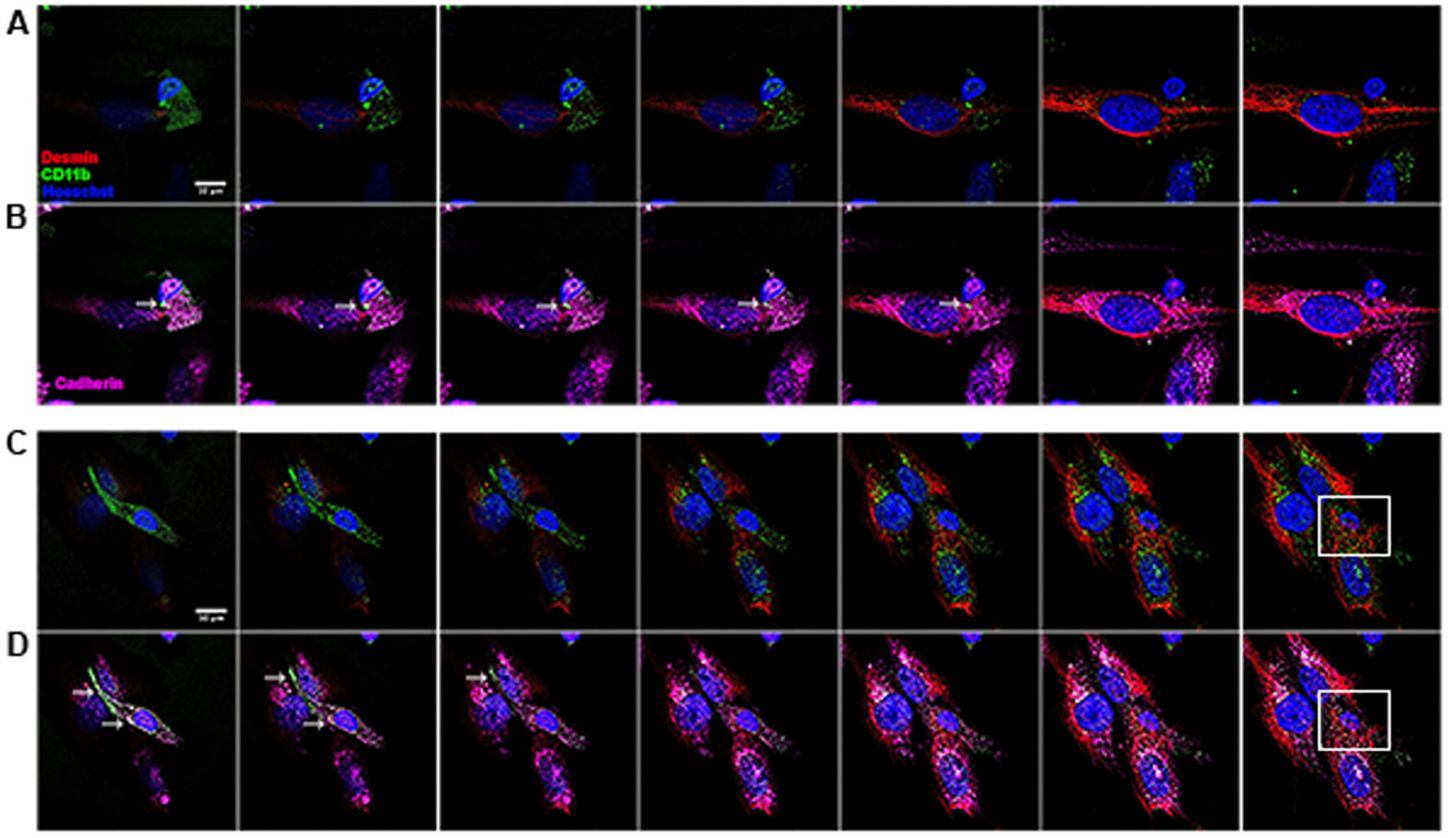
Confocal images of close contact between actDCs and myoblasts. Myoblasts were co-cultured with actDCs for 48 h, fixed and stained for image acquisition. Two different fields were captured to evaluate the close contact between the cells. **a** and **b** represent the *Z*-axis stacks of one field, whereas **c** and **d** represent the other field. Red desmin, green CD11b, blue nucleus, purple cadherin. The white arrow indicates the putative cell contact. The white square highlights the engulfment of the DCs by the myoblast. Data are representative of two different experiments

Our experiments were divided into three groups: (i) myoblasts alone; (ii) myoblasts incubated with iDC; and (iii) myoblasts incubated with iDC and LPS (called actDCs)^39^. We observed that the actDCs preferentially attached to the surface of the myoblasts (Fig. 1a, b). This was confirmed by transmission electron microscopy (Fig. 1b). The same results were observed when iDC were co-cultured with myoblasts (Supplementary Figure 1). This confirms that DCs and myoblasts are able to make close cell–cell contacts, providing evidence for possible cell-–cell communication and modulation. In order to determine if the interaction between these cells depends on specific adhesion molecules, the co-cultures were stained with antibodies against pan-cadherin, and cell specific markers such as CD11b (DCs) and desmin (myoblasts), and analyzed by confocal microscopy^40^. In stacked images obtained from the co-cultures of myoblasts and actDCs (Fig. 2), we observed a white focal staining (arrow, Fig. 2b, d), suggesting that both cell types establish a tight contact through the pan-cadherin proteins. In the Supplementary video 1 and 2 this interaction between the cells is even more evident. In Fig. 2c–d, the cytoplasmic prolongation of the activated dendritic cell onto the myoblast (arrow) is very clear, reinforcing the evidence of a tight cell-–cell interaction. Co-cultures of actDCs and myoblasts which had been differentiated into myotubes were also evaluated using confocal microscopy, and similar tight interactions were observed between the DCs and myotubes (Supplementary Fig. 1).

### actDCs induce HLA-ABC and HLA-DR expression on myoblasts

**Fig. 3:**
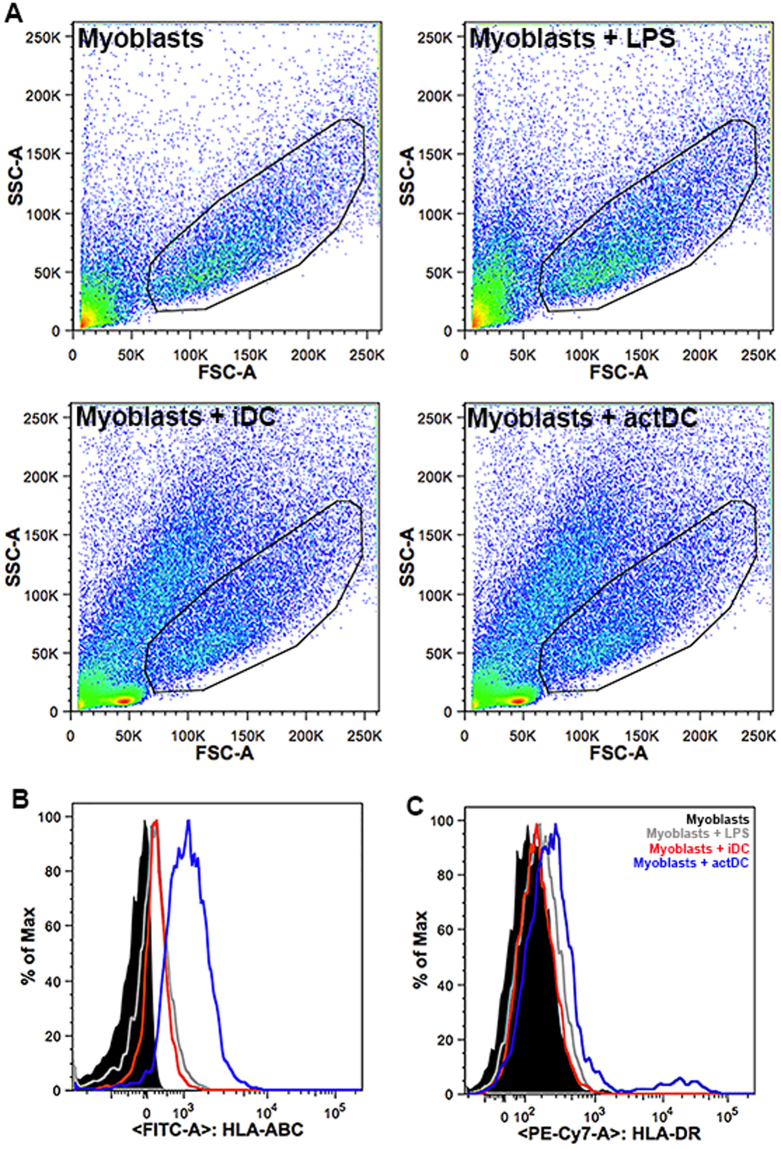
HLA-ABC and HLA-DR expression on myoblasts is increased during the co-culture with actDCs. Myoblasts were seeded in co-culture with iDC or actDCs, the cells were recovered, stained and analyzed by flow cytometry. **a** Dot-plots from myoblasts, myoblasts incubated with LPS (100 ng/mL), myoblasts incubated with iDC and myoblasts incubated with actDCs. The gated cells are CD56^+^ myoblasts. **b**, **c** reveal HLA-ABC and HLA-DR histograms, respectively, from myoblasts and DC co-culture, discriminated as black line myoblasts, gray line myoblasts + LPS, red line myoblasts + iDC, blue line myoblasts + actDCs. Data are representative of three different experiments

Previous studies of IIMs muscle biopsies demonstrated the expression HLA-ABC and HLA-DR on muscle cells^27,41–44^. We evaluated by flow cytometry HLA-ABC and HLA-DR expression in our iDC or actDCs and myoblast co-culture system. In Fig. 3a we demonstrated the myoblast-gated cells. After co-culture with actDCs (blue line), proliferating myoblasts exhibited an increased expression of HLA-ABC (Fig. 3b) and HLA-DR (Fig. 3c). Of note, not only is there an increase in the MFI of the DR staining on the largest myoblast population, but a small population of very bright cells appeared. However, when the myoblasts were co-cultured with iDC (red line) only HLA-ABC was induced (Fig. 3b). Interestingly, the activation of myoblasts by LPS (gray line) increased HLA-ABC expression in the same way as that observed in the co-cultures with iDC, but did not increase HLA-DR expression (Fig. 3b).

### Immature and actDCs promote proliferation and migration of myoblasts

**Fig. 4:**
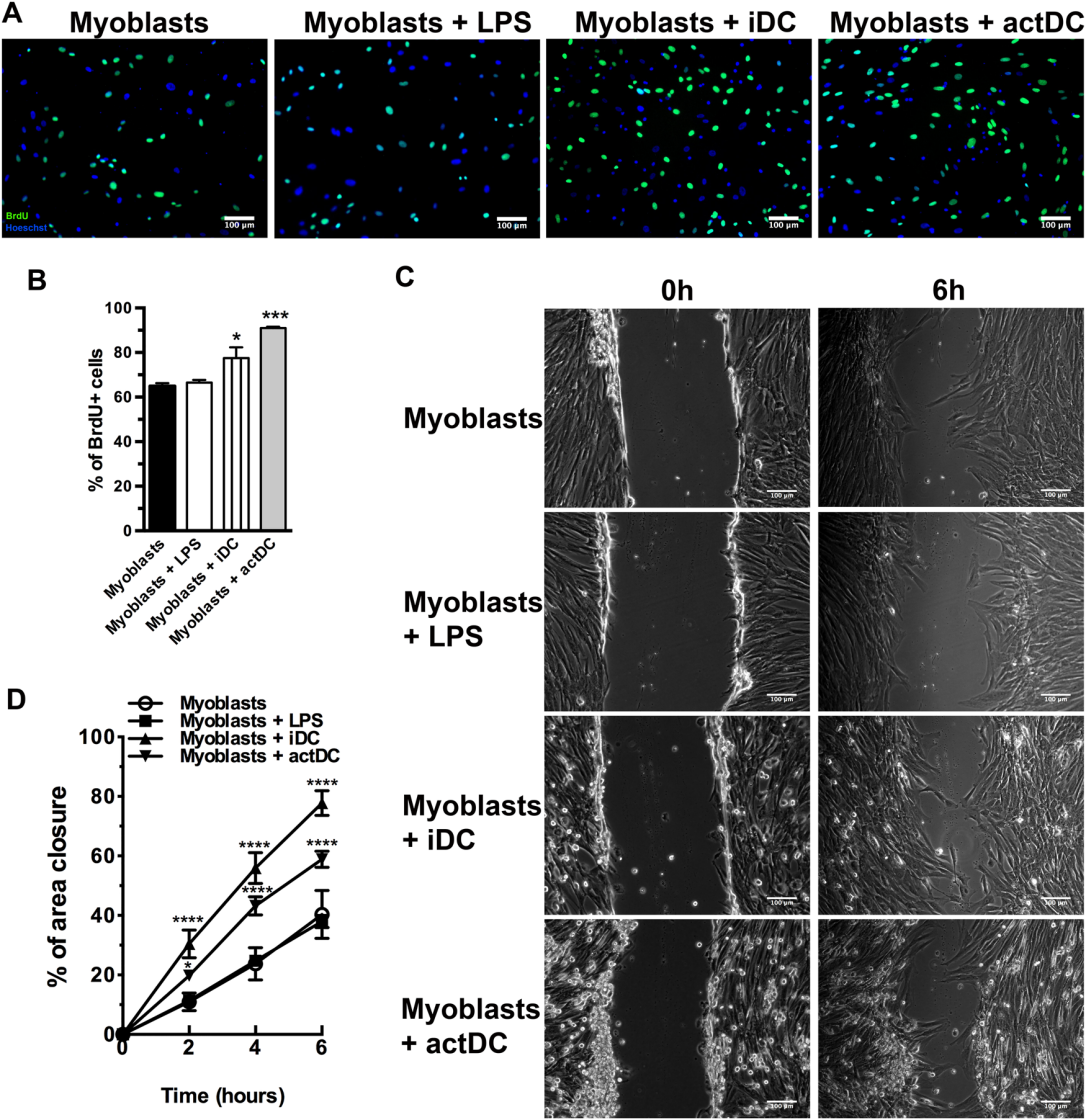
DCs incubation increase the proliferation and migration of myoblasts. **a** Myoblasts were co-cultured with iDC or actDCs for 48 h and the proliferation was evaluated by BrdU incorporation. **b** Quantitative analysis of the proliferation assay. The data are representative of three experiments. **c** Images representative of scratched areas from myoblasts alone or after incubation with LPS, or co-cultured with iDC or actDCs for 48 h. **d** The data are expressed as the percentage of the closed area. Data shown as means ± SE of duplicate wells and are representative of three different experiments. Bars represent 100 μm. *p < 0.05; ***p < 0.001, and ****p < 0.0001 compared to myoblasts

Myoblast proliferation and migration are two critical steps for muscle regeneration and modification of these parameters may result in an impairment of muscle repair. In our study, we observed an increase in proliferation when myoblasts were incubated with iDC or actDCs (Fig. 4a–b). One of our concerns was to assure that BrdU+ cells were only myoblasts and not DCs. In order to confirm this, the DC/myoblast co-cultures were double stained for CD11b and BrdU. As expected, CD11b+ DCs were BrdU− cells (Supplementary Fig. 2).

For myoblast migration, we performed an in vitro scratch assay and we analyzed the cell migration. After 6 h of incubation, myoblast migration was twice as fast as when cells were co-cultured with iDC or actDCs (Fig. 4c–d) as compared to myoblasts alone or myoblasts treated with LPS.

### Immature and actDCs inhibit myoblast differentiation

**Fig. 5:**
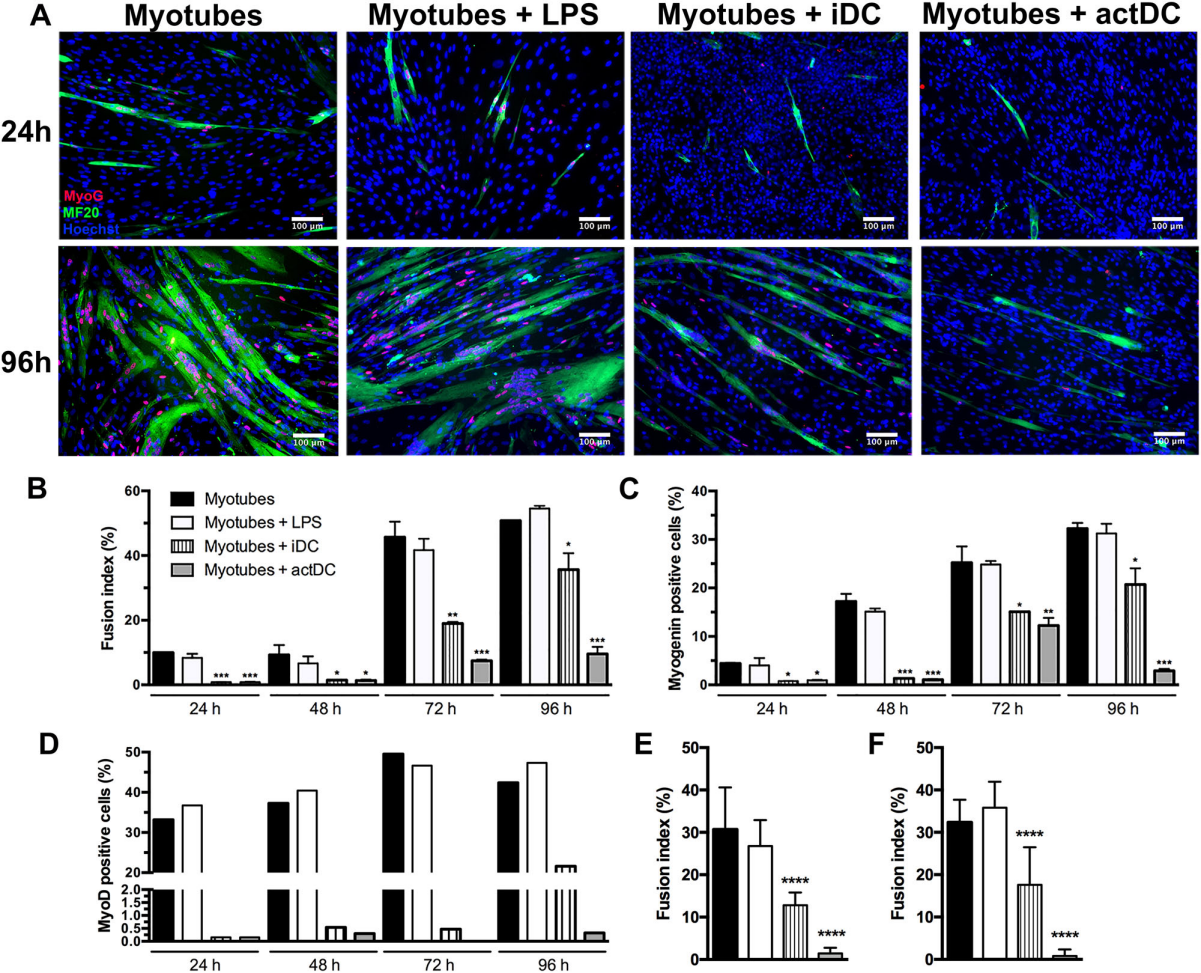
DCs inhibit myotube differentiation. **a** Immunostaining for myogenin (red), myosin heavy chain (green), and nuclei (Hoechst-blue) of myoblasts, myoblasts stimulated with LPS or co-cultured with iDC or actDCs after 24 and 96 h of incubation. The data represent three independent experiments. Bars represent 100 μm. **b**–**d** Graph representative of the fusion index, myogenin and MyoD expression presented in panel A evaluated at 24 to 96 h after incubation. **e**–**f** Fusion index of two different adult myoblast co-culture obtained from a 25 (**e**) and a 60-year-old (**f**) healthy donors evaluated at 96 h after incubation. Data shown as means ± SE of duplicates and are representative of three different experiments. *p < 0.05, **p < 0.01, and ***p < 0.001 compared to myoblasts

Another important step in muscle regeneration is the differentiation of myoblasts into new myotubes. In order to establish whether DCs modulate this step in vitro, the expression of myogenin and the fusion index were evaluated at 24, 48, 72, and 96 h of differentiation. We observed that in co-cultures of iDC with myoblasts, there was a reduction in myotube formation as shown in Fig. 5a. The inhibitory effect of actDCs on myotube formation was even more robust compared to iDC, since the differentiation was almost totally abolished at 96 h (Fig. 5a). This reduction in the fusion index of both iDC and actDCs co-cultures, was accompanied by a decrease in the number of myogenin and myoD positive cells (Fig. 5b–d and Supplementary Fig. 3A). Similar results were obtained using two different adult sources of human myoblasts (Fig. 5e–f).

In order to evaluate if the reduction in myotube formation in the presence of DCs was only the consequence of a delay in the differentiation, the differentiation was evaluated after longer periods time (120 and 144 h). In the presence iDC or actDCs, myoblasts did not differentiate even after 144 h of incubation and the low expression of myogenin was maintained (Supplementary Fig. 4). Further analyses were performed to identify if the cell cultures had lost their myogenic phenotype following incubation with DCs. To answer this question proliferating the cultures were stained with desmin, a specific marker for myoblasts and myotubes. After 48 h of incubation 80% of the cells in all groups were stained for desmin, confirming that they had maintained a myogenic phenotype (Supplementary Fig. 3B-C).

### Myoblasts and iDC or actDCs co-injection induced increased myoblast dispersion in an in vivo regeneration model

**Fig. 6:**
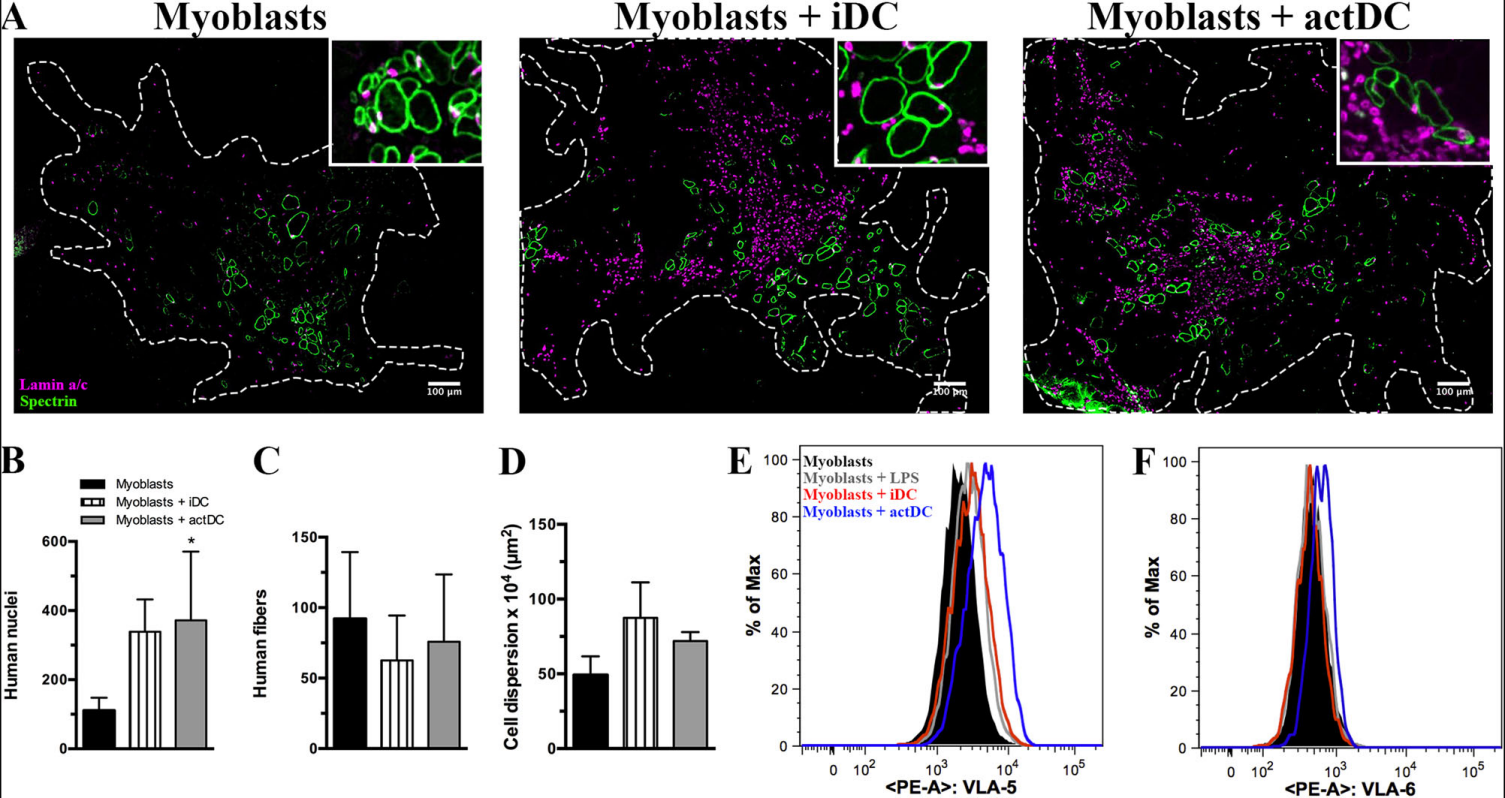
Co-injection of human myoblasts and DCs into the Tibialis anterior (TA) muscle pre-injured by cryolesion in Rag^-/-^IL2R^-/-^ mice. **a** Immunostaining for human specific lamin A/C (magenta) and spectrin (green) on muscle sections obtained 30 days after cryolesion of the tibialis anterior of Rag^−/−^IL2R^−/−^ mice. The injections of myoblasts, myoblasts plus iDC or myoblasts plus actDCs were made in 3–4 TA muscles per group. Bars represent 100 μm. The graphs show the number of human nuclei (**b**), human spectrin postive muscle fibers (**c**), and cell dispersion (**d**). The data represent one experiment. Myoblasts were seeded in co-culture with iDC or actDCs, and were recovered, stained and analyzed by flow cytometry. CD49e (**e**) and CD49f (**f**) histograms from myoblasts and DC co-cultures, discriminated as black line myoblasts, gray line myoblasts plus LPS, red line myoblasts plus iDC, and blue line myoblasts plus actDCs. Data are representative of 4 different TA muscles

In order to evaluate the behavior of human myoblasts when in contact with DCs in vivo, we used an experimental regeneration and transplantation model. Human iDC or actDCs were co-injected with human myoblasts into the tibialis anterior muscle (TA) of immunodeficient Rag2^−/−^/IL2rb^−/−^ mice following a cryolesion of the TA^45^. Figure 6a shows the presence of human fibers, revealed with a human specific antibody against lamin A/C (recognizing human nuclear protein) and against human spectrin (a sarcolemma protein) staining in the mouse TA, 30 days after injection (Fig. 6a upper inserts). Comparison of muscle regeneration following injections with myoblasts administered alone and co-injection of myoblasts and DCs shows an increase in the numbers of human nuclei and an increase in the dispersion of the human muscle fibers, although no difference was observed in the absolute number of human fibers that were formed (Fig. 6a–d). These confirm our in vitro results which predicted a greater migratory capacity, revealed by the dispersion, and proliferation, demonstrated by the increased number of nuclei. On day 30-post cell injection, a marked inflammatory infiltration was still observed in the regenerated muscle after DC co-injection (data not shown), which was not seen in the regenerated muscle after the administration of myoblast alone. These proliferation and migration data corroborate our in vitro results and provide strong evidence concerning the contribution of DCs to the pathophysiology of myositis.

### actDCs enhance the expression of VLA-5 and VLA-6 on myoblasts

In order to better understand the increased myoblast migration observed in vivo and in vitro, we evaluated the expression of integrin-type extracellular matrix receptors, which are crucial for cell-matrix contact and consequently cell migration^46^. VLA-5 and VLA-6 are membrane-bound integrins that bind fibronectin and laminin, respectively. These proteins are crucial for myoblast migration via myoblast–matrix protein interactions^47^, which favor myoblast dispersion. Consistent with our previous data, we found that iDC increased VLA-5 expression on myoblasts whereas actDCs enhanced both VLA-5 and VLA-6 expression, as revealed by flow cytometry (Fig. 6e, f). These results are in agreement with the in vivo data, suggesting that DCs may facilitate myoblast migration via increased integrin expression. These results reinforce the role of DCs, mainly actDCs, in modulating skeletal muscle cell mobility.

### Cytokine levels were increased after co-culture

In order to understand if the cell–cell contact is necessary for the role of DCs, differentiating myoblasts were incubated in the presence of conditioned medium (CM) obtained from iDC or actDCs. The CM obtained from actDCs inhibited both myotube formation and myogenin expression, whereas the CM obtained from the iDC co-culture had no significant effect on either fusion or myogenin expression (Supplementary Fig. 5A-C). Thus, although intimate cell–cell contact is present when DCs and myoblasts are co-cultured, secreted products from actDCs (possible cytokines) also play a role in inhibiting myotube formation independent of cell contact.

As described above, cytokines are critical for myoblast differentiation, and skeletal muscles express several cytokine receptors and release different types of cytokines^48,49^. To identify the mediators related to the DCs effects on myoblasts and myotubes, the cytokine and chemokine contents were evaluated in the supernatants from the co-cultures. During proliferation and differentiation, co-cultures of myoblasts or myotubes with iDC or actDCs released significant amounts of GM-CSF, IFNα2, IL-10, IL-13, IL-6, IL-8, CCL2, and TNFα. In differentiation conditions, GM-CSF levels were higher in the actDCs co-cultures; IL-13 level was higher and IL-6 level was lower in the iDC co-culture, all compared to the myotubes alone, myotubes plus LPS and iDC and actDCs single cultures. In proliferation, the level of IL-10 is higher in iDC co-culture and IL-13 is higher in actDCs co-culture, compared to myoblasts alone and myoblasts with LPS. It is interesting to highlight that some cytokines also presented lower levels in co-culture when compared to the level in the single culture of iDC or actDCs; for example, TNF in differentiation and IL-13 in proliferation. Also, we expected that LPS did not exert any direct effect on the myoblasts, but LPS increased GM-CSF and IL-8 levels in the supernatant of myoblasts alone in proliferation and differentiation (Table 1 A-B).

**Table 1:** Secreted cytokine levels in the supernatants from co-cultures of myoblasts and myotubes with DCs during proliferation and differentiation

A	GM-CSF (pg/mL)	IFNa2 (pg/mL)	IL-10 (pg/mL)	IL-13 (pg/mL)	IL-6 (pg/mL)	IL-8 (pg/mL)	CCL2 (pg/mL)	TNF-a (pg/mL)
Myoblasts	33.5 ± 2.1	13 ± 0.1	3.2 ± 0.1	3.2 ± 0.1	16079 ± 997.2	677 ± 36..8	9185 ± 559.7	11.6 ± 9.7
Myoblasts + LPS	198.5 ± 20.5	6.5 ± 0.7	3.2 ± 0.1	3.2 ± 0.1	15312.5 ± 888.8	5465.5 ± 522.6	10211 ± 1478.1	47.3 ± 25.5
Myoblasts + iDC	117.5 ± 14.9	16 ± 4.2	69 ± 8.5	8.6 ± 7.6	15811 ± 97.6	9093 ± 207.9	10872 ± 1020.1	146.5 ± 41.1
Myoblasts + actDCs	273 ± 28.3	9 ± 5.7	148.50 ± 6.4	34.5 ± 4.9	16134 ± 521.9	13960 ± 1233.2	10855.5 ± 565.9	44.8 ± 8
iDC	128.5 ± 20.5	3.1 ± 0.1	41 ± 4.2	100.5 ± 0.7	6.95 ± 0.5	9218.5 ± 1266.4	10744.3 ± 345.6	172.5 ± 75.77
actDCs	198 ± 22.6	7 ± 1.4	176 ± 14.1	93 ± 26.9	38.5 ± 13.3	13156 ± 2023.7	11283.5 ± 607.9	34.8 ± 4.1

B	GM-CSF (pg/mL)	IFNa2 (pg/mL)	IL-10 (pg/mL)	IL-13 (pg/mL)	IL-6 (pg/mL)	IL-8 (pg/mL)	CCL2 (pg/mL)	TNF-a (pg/mL)
Myotubes	17 ± 9.2	22.7 ± 3.2	3.2 ± 0.1	3.2 ± 0.1	4129.3 ± 69.5	1733.3 ± 355.6	9348 ± 383.3	3.2 ± 0.1
Myotubes + LPS	86.7 ± 23.6	24 ± 1.7	3..2 ± 0.1	3.2 ± 0.1	4922.3 ± 489.9	10940.7 ± 2241.6	9060.7 ± 1043.6	10.7 ± 4.2
Myotubes + iDC	148 ± 21.4	18.3 ± 2.1	127.3 ± 14.7	25.7 ± 2.9	879.7 ± 146.7	11003.3 ± 1276.9	8716 ± 1053	59 ± 11.4
Myotubes + actDCs	198.7 ± 30.1	16.7 ± 3.2	426 ± 17.4	19.7 ± 10.8	1447.3 ± 847.7	14099 ± 260.8	9513.3 ± 620.7	59.7 ± 8
iDC	3.2 ± 0.1	9 ± 4.6	98.7 ± 4.9	6.3 ± 2.1	190 ± 6.2	14914 ± 1358.7	7407.7 ± 409.9	61.3 ± 9.8
actDCs	32.7 ± 6.7	13.3 ± 1.5	682.3 ± 36.1	14.7 ± 5.5	870.3 ± 186.7	17411.7 ± 887.8	10517 ± 232.5	137.7 ± 14.2

Supernatants obtained from myoblasts, myoblasts stimulated with LPS, or co-cultured with iDC or actDCs, and supernatants obtained from iDC or actDCs single culture were analyzed by Luminex for GM-CSF, IFN-2α, IL-10, IL-13, IL-6, IL-8, CCL2, and TNF-α. The results in the tables are expressed as absolute numbers in pg/mL of supernatant, in proliferation (**A**) and in differentiation (**B**). Data are expressed as means ± SE and are representative of four different experiments.

## Discussion

The presence of DCs in the skeletal muscle has been previously demonstrated, but their exact role in the pathophysiology of IIM remains unknown^4,6,10–17^. We have demonstrated for the first time that DCs and myoblasts make close contacts when co-cultured in vitro and that DCs induced myoblast proliferation. We also observed in our co-culture system that both iDC and actDCs induced HLA-ABC and HLA-DR expression on myoblasts, as well as increased myoblast migration. However, this effect was dissociated from differentiation, as both iDC and actDCs impaired myotube formation.

We suggest that actDCs in our in vitro model, generated by the activation of iDC resemble those found in the muscle during inflammation, which express co-stimulatory molecules and release inflammatory cytokines^11,50^. Among the known subtypes of DCs, plasmocytoid DCs and myeloid DCs were found in the IIM muscle biopsies, nevertheless the amount of these cell populations in the affected muscles change according to the type of myositis. It is important to highlight that our in vitro data were developed with myeloid DCs (monocyte-derived dendritic cells–MDDC), which is the main dendritic cell differentiated from monocytes^6,7,11^.

The in vitro results were confirmed by the in vivo, where we observed an increase in proliferation as demonstrated by the significant increase in the number of human nuclei in the myoblast/DC-transplanted mice TA muscles. Moreover, in the same system we also found increased dispersion of the human myofibres within the host muscle tissue. In addition to the clear demonstration of tight contact between muscle cells and DCs, we showed that the co-culture supernatants also impaired myoblast differentiation and myotube formation.

Studies in the literature have also described HLA-ABC and HLA-DR expression on muscle cells during inflammation^41,44,50,51^. The damage-based mechanism of necrosis in skeletal muscle tissue is characterized by an upregulation of HLA-ABC on muscle fibers that prompted those fibers to be recognized by CD8+ T cells, which attack and invade the HLA-ABC+ muscle fibers in DM patients^25^, by releasing several mediators such as granzyme-B and perforin-1^52^. The importance of HLA-ABC expression on muscle cells during the disease course is described by Nagaraju et al. who engineered a mouse model overexpressing MHC-class I and observed that these animals develop several features of IIM, such as muscle weakness and the expression of type I IFN inducible genes^51^. In our study, we have demonstrated that DCs play a role in this process by increasing HLA-ABC expression.

The in vivo experiments showed that myoblasts migrate more in the presence of actDCs resulting in an increased dispersion of the human fibers. In accordance, Bencze et al. recently demonstrated that proinflammatory macrophages enhanced the regenerative ability of human myoblasts in the same transplantation model. Interestingly, the M1 inflammatory macrophages, co-injected with myoblasts, switch to M2 macrophages 10 days after injection, supporting the idea that limited inflammatory environment is important for myoblast proliferation and an anti-inflammatory environment supports differentiation^53^. Thus, we demonstrated a negative impact of inflammation and actDCs on regeneration in the same mouse model. Moreover, the increased number of human nuclei in the healed muscle tissue 30 days after myoblasts and DC co-injection is in agreement with the hypothesis that in vitro actDCs induce myoblast proliferation and inhibit differentiation. Also, our results agree with the previous one, which showed that inflammatory environment impairs differentiation^2,4,7^. Although the model we use is a xenogeneic one, with human myoblasts transplanted into a mouse, xenogeneic reactions are not present as the transplanted host is immune-deficient and lacks T cell. So, the effects of DCs on myoblast differentiation are probably due to a direct effect on the myoblasts, in accordance to our in vitro data. It should be noted that there was also no allogeneic reaction in our in vitro model, as our experimental conditions was in the absence of T cells, confirming that the interaction observed between DCs and myoblasts was independent of allorecognition.

Previous studies have shown that inflammatory molecules affect the expression of MRF’s, such as Pax7, MyoD, and myogenin^54^. Cytokines such as TNFα, IL-6, and IL-1α exert inhibitory effects on myogenesis and alter the expression of MFR’s in skeletal muscle tissue and this may explain the disturbance in muscle regeneration, which has been observed in sIBM^55^. These mediators are released during inflammation and regeneration by all cell types, including leukocytes, myoblasts, and myotubes^56,57^. Accordingly, DC-derived conditioned medium was able to partially mimic the effect observed in DC/myoblast co-cultures. In fact, the effect of cytokines on myotube formation is both time and concentration dependent, low levels of cytokines in the early phase of regeneration promotes myoblast proliferation and myotube formation, while in a chronic phase or in the case of high levels of cytokines, the differentiation is inhibited^55^. Furthermore, there are few data in the literature that consistently support the role of cytokines during muscle differentiation. It has been demonstrated that IL-4/IL-13 signaling promotes proliferation of fibro/adipocyte progenitors and myogenicity^58,59^. Surprisingly, it was also demonstrated that IL-10 contributes to muscle growth, as it plays an important role in the switch of macrophages from a M1 to M2 phenotype in injured muscle^60^. On the other hand, it has already been showed that TGFβ impaired myoblast proliferation and myotube formation^61^. Moreover, chronically elevated production of IL-6 promotes skeletal muscle wasting, but low concentration of IL-6 promotes repair and skeletal muscle regeneration^62^. In our model, we demonstrated that myoblasts stimulated with LPS only had an effect when incubated with DC co-culture, reinforcing the effect of secreted cytokines in skeletal muscle cells. In this work, during co-culture with iDC or actDCsactDCs, we observed an increase of some cytokines production in both co-cultures, though our data were not enough to delineate one or a group of cytokines responsible to the effects attributed to the DCs.

The importance of cytokine production in skeletal muscle was highlighted by our group demonstrating that IL-4 and IL-13 play a critical role in myotube integrity, since auto-antibodies, such anti-SRP and anti-HMGCR, induced myotube atrophy mediated by a reduction of IL-4 and IL-13 levels in the supernatant of confluent human myoblasts8. We also provided evidence in vitro that adipocytes from visceral tissue induced inflammation and atrophy due to reduced expression of muscle proteins, and that IL-1β and IL6 are important contributors^63^. These data strongly corroborate the idea of a cross-talk through cytokine released from muscle cells and other infiltrating cells as fibro/adipocytes progenitors (FAPs) and leukocytes, which play a critical role in muscle dysfunction.

Our findings, together with the literature, bring some evidence that DCs may play a significant role in activating myoblasts and myotubes, consequently promoting a cytokine rich environment that may trigger signaling pathways, such as NFκB and MAPK, that are involved in myoblast differentiation and atrophy^64–66^. Nevertheless, we cannot ignore the close cell–cell contact of DCs and myoblast, which may trigger the cytokine effect.

In conclusion, we have demonstrated that there is an important interaction between myoblasts and DCs during inflammation. Despite the fact that an acute inflammation should help regeneration, a persistent inflammation may cause muscle atrophy and impair wound healing^67–70^. Therefore, we suggest that actDCs in muscle tissue interfere, both directly and indirectly, in myoblast activation, proliferation, and differentiation in the early phase of myositis, re-feeding the inflammation, probably increasing antigen presentation and consequently impairing myotube formation. Our work contributes to a better understanding of myositis and hopefully a step further to its therapy.

## Material and methods

All the procedures were in accordance with the French legislation on ethical rules. Supplementary information is available at Cell Death and Differentiation’s website.

### Myoblast cultures

All data were performed using a human myoblasts isolated from quadriceps muscle biopsies of 5-day-old infant with no neuromuscular disorders, as previously reported^71^. In order to prove that this cell is a reliable model, we used two other sources of human myoblast, which were quadriceps muscles from 25 and 60-year-old healthy donors. Myoblasts were cultivated in growth medium (DMEM [Gibco, Carlsbad, CA, USA], 50 µg/ml gentamicin [Gibco], 20% [v/v] fetal bovine serum [FBS, Gibco]) and the myogenic purity of the populations was monitored by immunocytochemistry using desmin (Dako, Golstrup, Denmark) as marker. Differentiation was induced at confluence by replacing the growth medium with serum-free DMEM supplemented with 50 µg/mL gentamycin and 10 µg/mL of human insulin (Sigma Aldrich, St Louis, MO, USA). Myotube formation was assessed by fusion index. The fusion index was calculated as the ratio of the number of nuclei inside myotubes to the number of total nuclei ×100 at day 4 of myogenic differentiation, using three independent fields.

### Purification of DCs

The DCs were generated from human peripheral blood^37^ (buffy coat) from healthy adult donors. The buffy coat was submitted to Ficoll (Sigma) gradient. The mononuclear cells were collected by centrifugation and incubated in 75 cm^2^ culture flasks (Falcon, Becton Dickson, San Diego, CA, USA, 1,5 × 10^8^ cells/flask) with serum-free RPMI medium (Gibco) for 1 h at 37 °C in 5% CO_2_. Floating cells were removed by PBS wash and then incubated with RPMI (Gibco) supplemented with 10% FCS, 50 µg/mL GM-CSF (R&D Systems, Minneapolis, MN, USA) and 50 µg/mL IL-4 (R&D Systems). On day 3, half of the medium was recovered, centrifuged, and the cells were resuspended in the same volume with RPMI supplemented with 50 µg/mL GM-CSF and 50 µg/mL IL-4. On day 6, floating DCs were recovered and submitted to negative selection kit according to the manufacture instructions (Miltenyi Biotec, Bergisch Gladbach, Germany). The DCs were labeled with CD11c or CD11b and the purity were analyzed by flow cytometry. In all experiments, DCs purity was 80%. These steady state DCs were called iDC. In order to generate mature DCs (inflammatory/actDCs) that express high levels of HLA-DR, co-stimulatory molecules and secreting inflammatory cytokines^72^, the co-culture of iDC with myoblasts was performed in the presence of lipopolysaccharide (LPS, E. coli serotype O26:B6; Sigma Cat. No. L2762) throughout the incubation time^39,73^.

### Co-culture assays

In all experiments, the myoblast-DC co-cultures were made in 1:3 ratio because myoblasts are proliferating cells^74^ and differentiated DCs are not^38^, and DCs are much smaller than myoblasts (DCs are the same size as the myoblast nucleus). In proliferation, 1.5 × 104 myoblasts were co-cultured with 4.5 × 104 DCs with or without of LPS (100 ng/mL) in 24-well plates for 48 h at 37 °C in 5% CO2. In differentiation assay the 5 × 104 myoblasts per well were cultured in 24-well plates overnight at 37 °C in 5% CO2 or until confluence. Then, the medium was switched for differentiation medium and 1.5 × 105 DCs with or without LPS (100 ng/mL) were added per well for 96 h at 37 °C in 5% CO2. LPS was present during the incubation time (48 or 96 h).

### Electron microscopy

The co-cultures grown on Thermanox^™^ cover slips were fixed with 2% glutaraldehyde, 2% paraformaldehyde. After 2% OsO_4_ post-fixation, they were gradually dehydrated with acetone, including 1% uranyl in 70% acetone step, and finally embedded in Epon®. A total of 70 nm sections stained with uranyl acetate and lead nitrate were observed in a Philips CM120 electron microscope equipped with a SIS Morada camera (Philips, The Netherlands).

### Immunofluorescence and confocal microscopy

We performed staining for myosin heavy chain (MF20 – DSHB, hybridome), myogenin (Dako), myoD (Dako), desmin (Dako), and pan-cadherin (Dako). The co-culture in differentiation was fixed with 4% paraformaldehyde at 24, 48, 72, and 96 h and stained with the specific primary antibody. The primary antibody was revealed using conjugated secondary antibody and the nuclei were stained with Hoechst (Sigma). The images were obtained with inverse fluorescent microscopy Axio Observer.A1 and AxioCam Camera (Carl Zeiss, Germany).

For confocal microscopy analysis, the co-cultures were grown on cover slips in 24-well plates for both proliferation and differentiation. After incubation, the cells were fixed with absolute ethanol for 10 min, and then stained for CD11b (Abcam, Cambridge, United Kingdom), desmin, myosin heavy chain (MF-20 – DSHB, Iowa, USA), and cadherin (Abcam). Specific primary antibody binding was revealed using conjugated coupled specific secondary antibody. The stacked images were analyzed with inverse confocal microscopy AOBS SP2 Leica (Leica Microsystems, Wetzlar, Germany). The video with the sequential stacks can be visualized in supplementary video 1 and video 2.

### Quantification of the fusion index, myogenin, and myoD

Myogenin and myoD expression was measured by counting the number of positive nuclei for myogenin or myoD and divided by the total number of myoblast nuclei in each image. In order to exclude the DC nuclei, we used the total number of myoblast nuclei obtained from the images of myoblast alone group. It is also worth noting that DC nuclei are much smaller and has different morphology as compared to myoblast nuclei. The data were determined by counting more than 1500 nuclei per group, and were expressed as percentages. The counting was performed using the Cell Profiler® software and ImageJ® software.

### Quantification of cytokines

The co-cultures were grown in 96-well plates with the same ratio of myoblasts over DCs in proliferation and differentiation. The cytokine release was evaluated by Luminex methodology (Invitrogen, Carlsbad, CA, USA), according to manufacture’s instructions, using the supernatants from 48 and 96 h co-cultures, respectively. The panel of inflammatory cytokines used was for GM-CSF, IL-10, IL-13, IFN-2a, IL-6, IL-8, CCL2, and TNF-α. The data were acquired with Luminex® xMAP® System and the analysis was with Luminex® 200TM software.

### Flow cytometry

Co-cultures of myoblasts with immature or actDCs, or myoblasts alone in proliferation, were incubated in 150 cm^2^ flasks for 48 h in the same ratio as described above. Then the cells were scraped, recovered, and stained with fluorochrome labeled specific antibodies: CD56-APC, HLA-ABC-FITC, HLA-DR-PE-Cy7, VLA-5-PE, and VLA-6-PE (Becton-Dickinson Bioscience, San Jose, CA, USA). The specific staining was evaluated in the CD56 positive cell gate. The data were acquired in using a FACS Aria (Becton-Dickinson) and the analyses were carried out using the FlowJo® Software.

### Cell proliferation

A volume of 10 μg/mL of BrdU (Thermofisher, Boston, MA, USA) was added in the last 24 h of incubation into the proliferating co-cultures. The cells were then fixed with paraformaldehyde 4% and stored at 4 °C. Before performing the BrdU staining, the epitopes were exposed with 2 M HCl incubation for 30 min, and then with 50 mM NaCl + 10 mM Tris HCl, pH 7.5, for 30 min. After that, the specific primary antibody was applied for 60 min and specific labeling was revealed using a conjugated secondary antibody. The nuclei were stained with Hoechst. The proliferation was calculated by counting the number of BrdU^+^ nuclei and dividing by the total number of nuclei in each well, using two wells per group. More than 1500 nuclei per condition were counted and the data were expressed as the percentage of BrdU^+^ cells. In order to discard DCs proliferation, these cells were stained for CD11b expression

### Cell migration

To analyze the myoblast migration, the co-cultures were performed in 6-well plate keeping the 1:3 myoblast/DC ratio. The cells were incubated for 48 h at 37 °C in 5% CO^2^, until reaching confluence. Then the supernatants were stored at 37 °C in 5% CO_2_ while the monolayers were treated with mitomycin C 50 µg/mL (Sigma) for 1 h 37 °C in 5% CO_2_. After that, the cell monolayers were scratched and washed with PBS at 37 °C. The previous supernatants were returned to the 6-well plate, respectively, to each condition. The images were obtained at 0, 2, 4, and 6 h using the Zeiss inverted microscopy Axio Observer.A1 and AxioCam Camera (Carl Zeiss, Germany).

### Cell differentiation in conditioned medium

iDCs were incubated with or without LPS for 48 h in 24-well plates. Then, the supernatant was recovered and centrifuged at 400 g for 10 min at 4 °C. The conditioned medium was added to confluent cells cultivated for 96 h at 37 °C in 5% CO_2_. The myotube formation was analyzed as described above (fusion index).

### In vivo experiments

Rag2^−/−^IL2rb^−/−^ mice, aging 8–12 weeks, double knockout mice for Rag2 and IL2R genes were used for the regeneration model^45^. The mice were kept in a cycle of light and dark, and had free access to food and water. The detailed surgical procedures are provided in^75^. Mouse TA muscles were excised for histological analysis. Cryosections from TA were used to count human nuclei and fibers and to analyze human cell dispersion with the human specific antibodies: lamin A/C (Abcam), spectrin (Leica Biosystems). To visualize the nuclei, the sections were mounted in vectashield mounting medium with dapi (Vector laboratories). Pictures were taken with a Microscope Nikon AZ100M.

### Statistical analysis

The experiments were performed at least three times, using DCs from different human donors. Statistical analyses were performed using GraphPad Software (San Diego, CA, USA). Values are expressed as means ± standard error of triplicates from one experiment performed with the same DC preparation. Comparisons were carried out using two-way analysis of variance (ANOVA) followed by Bonferroni tests. Differences were considered significant when *p*-value was <0.05.

## Electronic supplementary material

Suppemental material

Video 1

Video 1
