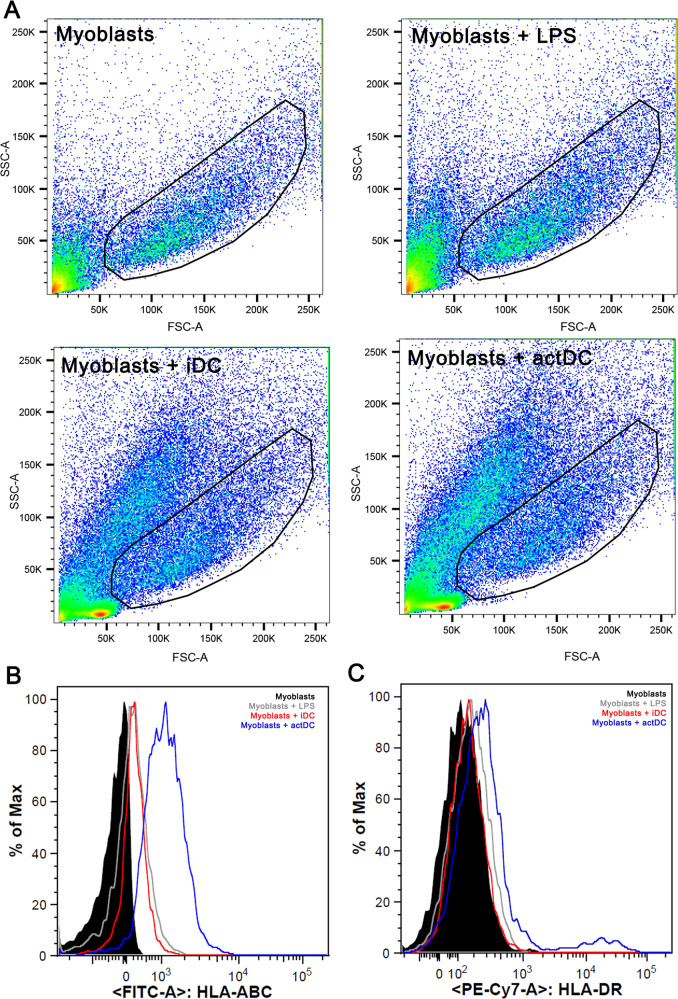# Correction: Activated dendritic cells modulate proliferation and differentiation of human myoblasts

**DOI:** 10.1038/s41419-022-05278-7

**Published:** 2022-09-22

**Authors:** Leandro Ladislau, Débora M. Portilho, Tristan Courau, Alhondra Solares-Pérez, Elisa Negroni, Jeanne Lainé, David Klatzmann, Adriana Bonomo, Yves Allenbach, Olivier Benveniste, Ingo Riederer, Wilson Savino, Vincent Mouly, Gillian Butler-Browne, Claudia F. Benjamim

**Affiliations:** 1 Institute of Biophysics Carlos Chagas Filho, Federal University of Rio de Janeiro, Rio de Janeiro, Brazil; 2 Institut de Myologie, INSERM U974, Sorbonne Université, Paris, France; 3 Immunology-Immunopathology-Immunotherapy, INSERM U959, Sorbonne Université, Paris, France; 4 Laboratory on Thymus Research, Oswaldo Cruz Institute, Fiocruz, Rio de Janeiro, Brazil; 5 National Institute of Science and Technology on Neuroimmunomodulation, Rio de Janeiro, Brazil

Correction to: *Cell Death and Disease* 10.1038/s41419-018-0426-z, published online 10 May 2018

The original version of this article unfortunately contained an error in figure 3. The authors apologize for the mistake. The corrected figure can be found below.