# Supplementary material for: Activated dendritic cells modulate proliferation and differentiation of human myoblasts
Source: Cell Death Dis. 2018 May 10;9(5):551. doi: 10.1038/s41419-018-0426-z (PMC5945640; doi:10.1038/s41419-018-0426-z)
Supplement: Supplementary file 1 — Suppemental material [file 41419_2018_426_MOESM1_ESM.docx]

Supplementary information

Supplementary Videos 1 and 2. Confocal sequential images of close contact between actDC and myoblasts. Myoblasts were co-cultured with actDC for 48 hours, fixed and stained for image acquisition. In red – desmin; green – CD11b; blue – nuclei; and purple – cadherin. The image sequence represents Z-axis stacks of one field. Data are representatives of two different experiments.


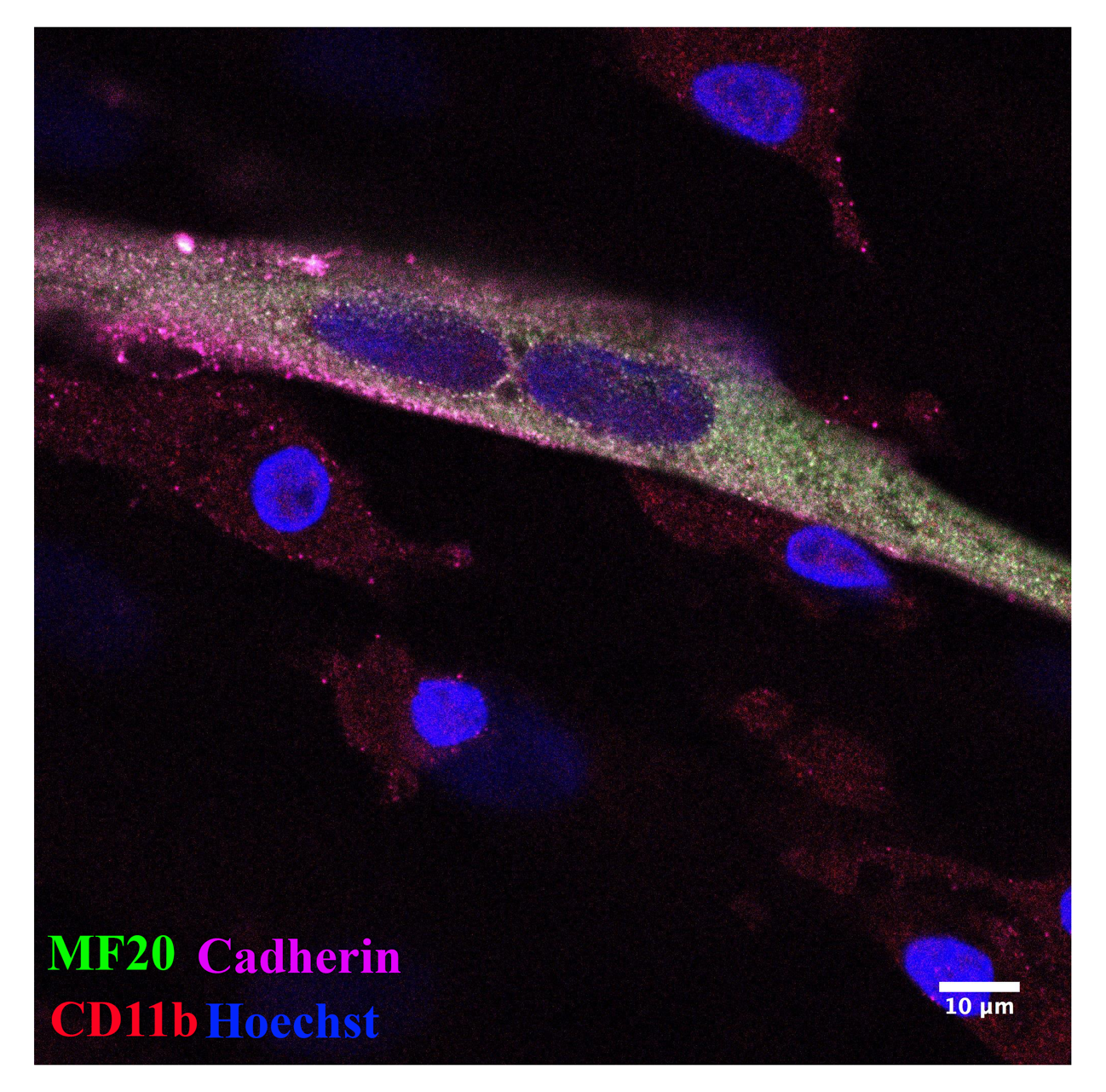


Supplementary Figure 1. Confocal images of close contact between actDC and myotubes. Myotubes were co-cultured with actDC for 48 hours, fixed and stained for image acquisition. Three different fields were captured to evaluate the close contact between the cells. **A, B** and **C** represent different microscopic fields. In red – CD11b; green – MF20; blue – nuclei; and purple – cadherin. Data are representative of two different experiments.


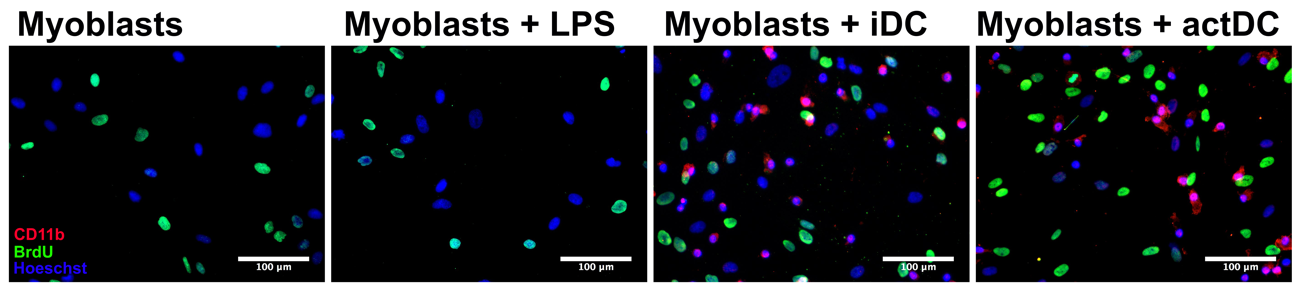


Supplementary Figure 2. Co-culture incubation induces myoblast proliferation of myoblasts, but not DCs. Myoblasts were co-cultured with iDC or actDC for 48 hours and the proliferation was evaluated by BrdU incorporation. The cells were fixed and stained for images acquisition after co-culture. In red – CD11b; green–BrdU; and blue – nuclei. Bars are representative of 100 μm. Data correspond one experiment.


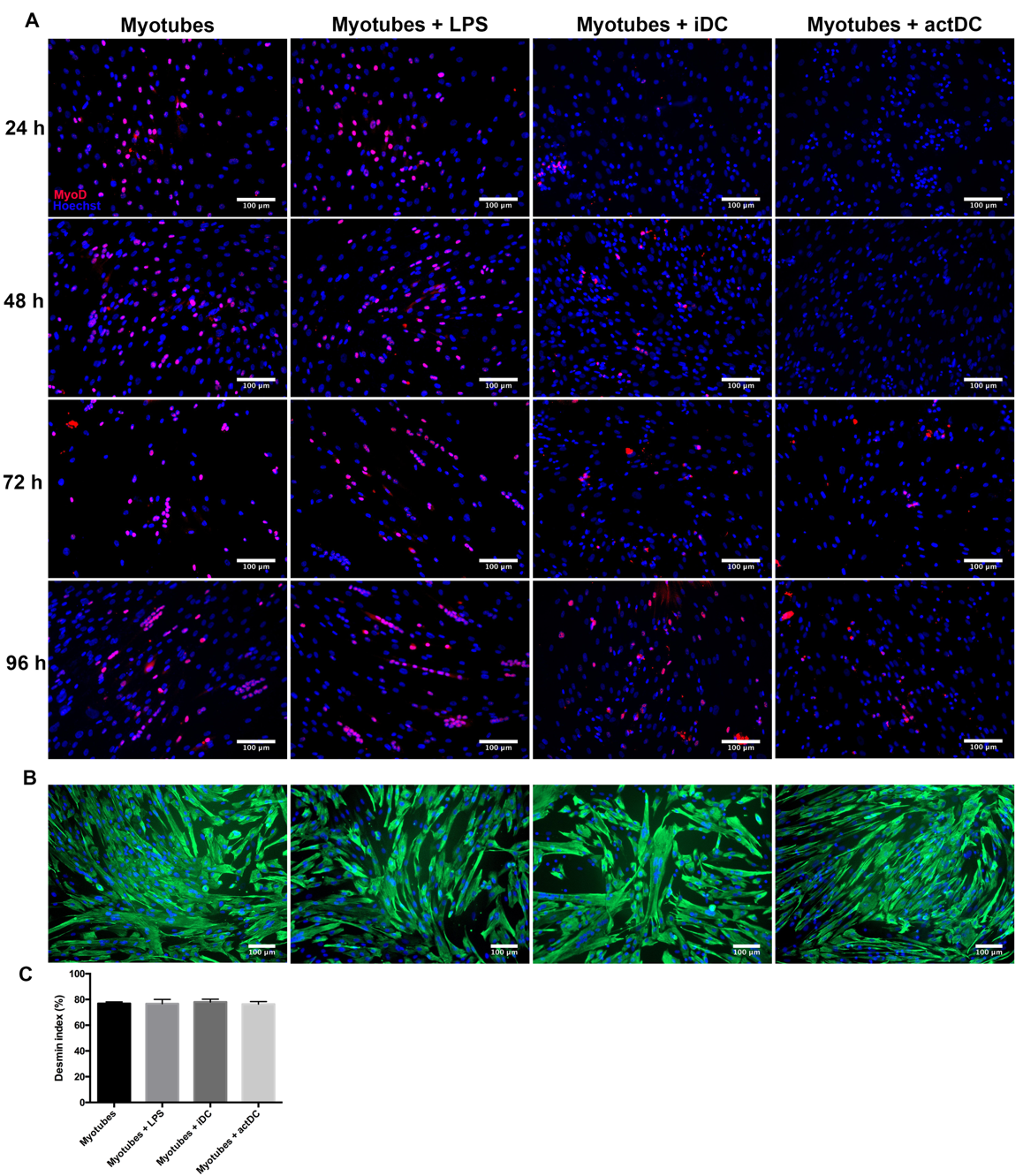


Supplementary Figure 3. Immature and activated DCs inhibit myoD expression and our human myoblasts present high myogenicity. (**A**) Immunostaining for myoD (red), and Hoechst (blue) of myoblasts, myoblasts stimulated with LPS, or co-cultured with iDC or actDC at 24 to 96 hours of incubation during differentiation. (**B**) Immunostaining for desmin (green) and Hoechst (blue) at 48 hours in differentiation assay. **(C)** Desmin index presented in panel B. Data correspond to one experiment. Bars represent 100 μm.


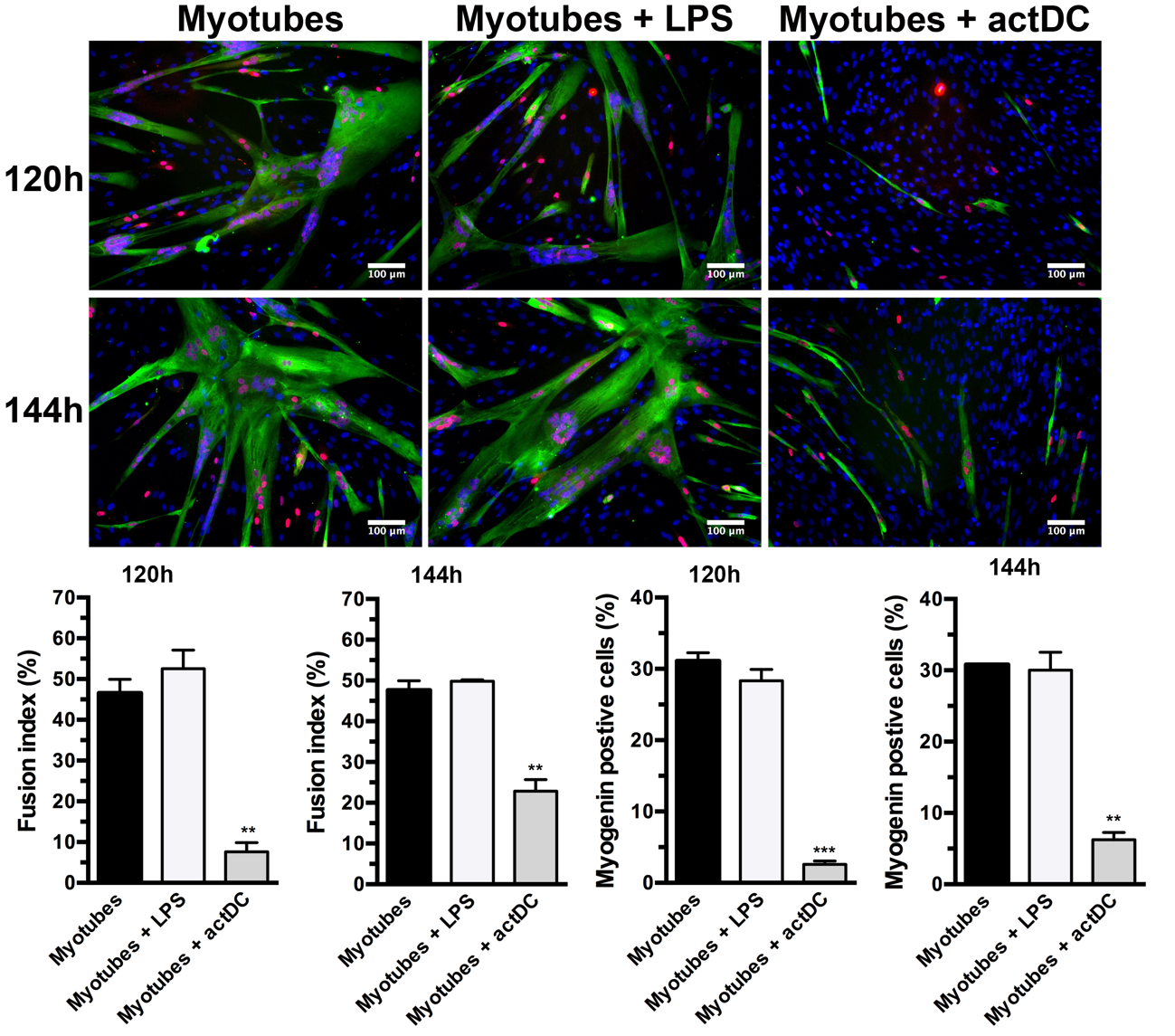


Supplementary Figure 4. iDC and actDC inhibit myotube formation at 144h. Immunostaining for myogenin (red), myosin heavy chain (green) and nuclei (Hoechst-blue) of myoblasts, myoblasts stimulated with LPS, or co-cultured with actDC at 120 and 144 h of incubation. The graphs show fusion index and the quantification of myogenin positive cells at 120 and 144 h of incubation. Data shown as means ± SE of duplicates and are representative of three different experiments. Bars represents 100 μm. **p< 0.01 and ***p< 0.001 compared to myoblasts.


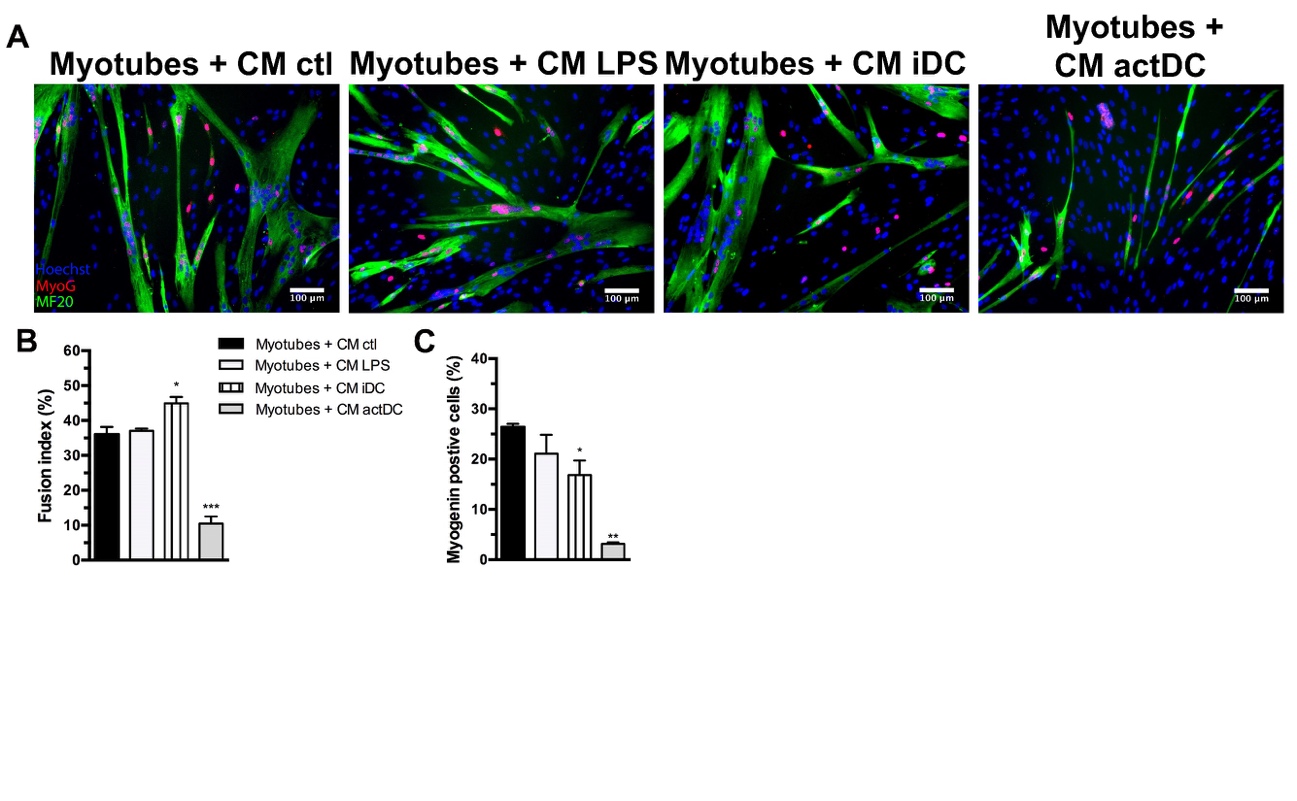


Supplementary Figure 5. Conditioned medium from actDC inhibit myotube differentiation. (**A**) Immunostaining for myogenin (red), myosin heavy chain (green) and nuclei (Hoechst-blue) of myoblasts incubated for 96 hours with conditioned medium obtained from myoblasts, myoblasts stimulated with LPS, or co-cultured with iDC or actDC. The graphs show fusion index (**B)** and myogenin expression (**C**) at 96 h incubation. Data shown as means ± SE of duplicates and are representative of three different experiments. Bars represents 100 μm. *p< 0.05, **p< 0.01 and ***p< 0.001 compared to myoblasts.


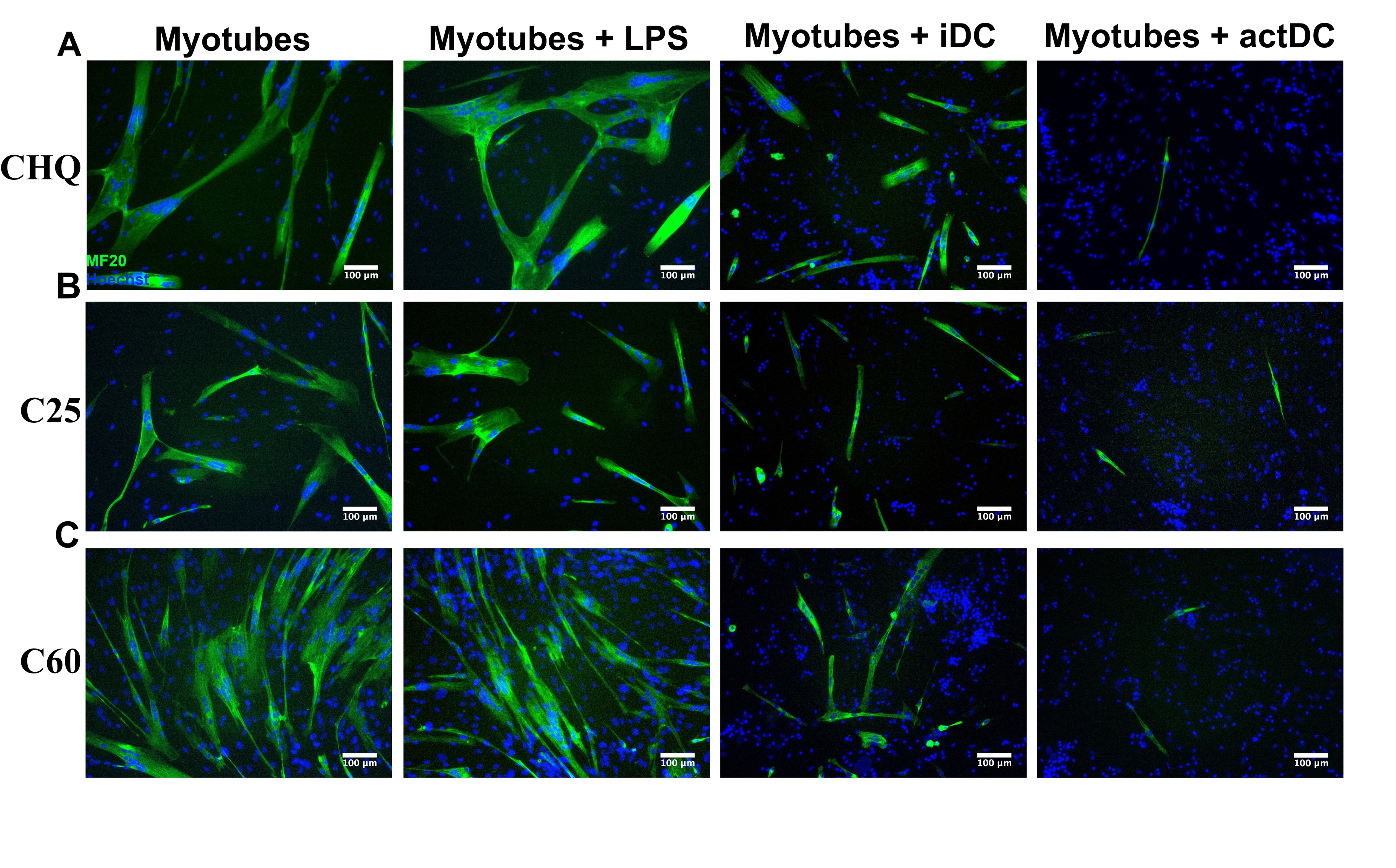


Supplementary Figure 6. DCs inhibit myotube formation of two different adult human myoblast cultures. Immunostaining for myosin heavy chain (green) and nuclei (Hoechst-blue) of myoblasts, myoblasts stimulated with LPS, or co-cultured with iDC or actDC at 24, 48 and 72 h of incubation. Myoblasts were obtained from the quadriceps muscles of donors aged 5 days (A), 25 years (B), and 60 years (C). Bars represent 100 μm.


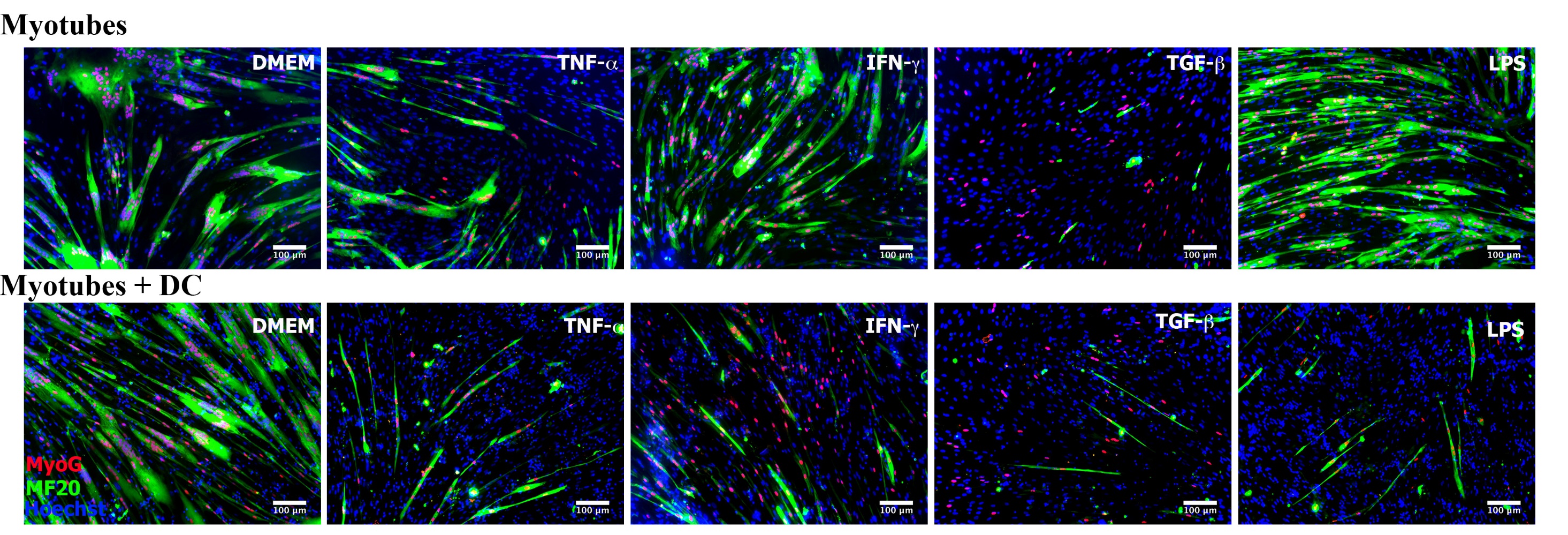


**Supplementary Figure 7.** **Inflammatory cytokines and LPS inhibit myotube differentiation.** Immunostaining for myogenin (red), myosin heavy chain (green) and nuclei (Hoechst-blue) of myoblasts alone (A) or co-cultured with iDC in the presence of TNF-α (50 ng/mL), IFN-γ (10 ng/mL), TGF-β (10 ng/mL), or LPS (100 ng/mL) for 96 h incubation. DMEM is the medium used as a control. Bars represent 100 μm. The data represent one experiment.
